# Biogenic iron nanoparticles as a new priming solution to improve seed germination and vigor in pigeonpea (*Cajanus Cajan* L.)

**DOI:** 10.1186/s12870-025-08044-x

**Published:** 2026-01-03

**Authors:** Akshay Kumar Kurdekar, B. K. Desai, Manjanagouda S. Sannagoudar, Hanamant M. Halli, B. G. Koppalakar, Pandit S. Rathod, Sharanagouda Hiregoudar, N. L. Rajesh, M. R. Umesh, Vishwanatha Sakra Naik

**Affiliations:** 1 Department of Agronomy, University of Agricultural Sciences, Raichur, Karnataka India; 2 ICAR-National Institute of Seed Science & Technology, Regional Station, Bengaluru, India; 3 ICAR-National Institute of Abiotic Stress Management, Baramati, India; 4 Department of Agronomy, College of Agriculture, Kalaburagi, Karnataka India; 5 Department of Processing and Food Engineering and Centre for Nanotechnology, University of Agricultural Sciences, Raichur, Karnataka India; 6 Department of Soil Science and Agricultural Chemistry, University of Agricultural Sciences, Raichur, India

**Keywords:** Green chemistry, Nano iron, Pigeonpea and green synthesis

## Abstract

This study aimed to evaluate the effectiveness of biogenic iron nanoparticles as a novel priming solution to enhance seed germination and vigor in pigeonpea (*Cajanus cajan* L.). Iron nanoparticles synthesized through green methods have received increasing scientific attention for their agricultural applications, particularly in enhancing seed germination and plant growth. In this study, eco-friendly, plant-based extracts were used to synthesize iron nanoparticles, explored as a nutritional supplement to boost pigeonpea seed germination. The green synthesis involved reducing iron salts using plant extracts, serving as both reducing and stabilizing agents. Characterization using UV-vis spectrophotometry, dynamic light scattering (DLS), and scanning electron microscopy (SEM) confirmed the nanoparticles’ nano-scale dimensions, stability, and crystalline structure. Seed priming with green-synthesized nanoparticles at an optimal concentration of 50 ppm significantly improved germination (97.76%), root and shoot lengths, and seedling dry weight, while reducing abnormal seedlings and dead seeds. These effects were attributed to enhanced bioavailability, improved enzymatic activity, and efficient nutrient delivery during germination. The results highlight the potential of green-synthesized iron nanoparticles as a effective seed priming agent in pigeonpea cultivation.

## Introduction

Pigeonpea (*Cajanus cajan* L.) is a vital legume crop in semi-arid and tropical regions, valued for its protein-rich seeds and role in sustainable agriculture through biological nitrogen fixation. Despite its agronomic importance, pigeonpea cultivation often faces challenges such as poor and uneven seed germination, leading to suboptimal plant stand and reduced productivity. Enhancing early seedling vigor is critical to improving crop establishment, resilience, and overall yield [1].

In recent years, nanotechnology has revolutionized multiple sectors, including agriculture, offering innovative approaches to address challenges in food security and sustainability. In the realm of crop production, nanotechnology applications such as nanoparticle-based fertilizers, pesticides, and growth promoters have emerged as transformative tools. Among these, green-synthesized nanoparticles have gained substantial attention due to their eco-friendly and cost-effective characteristics [2].

Unlike conventional chemical methods, green synthesis involves the use of biological agents such as plant extracts, microorganisms, or other natural sources. This process minimizes the environmental risks associated with nanoparticle production and leverages bioactive compounds in the biological agents to enhance the functional properties of the nanoparticles [3].

Iron (Fe) is a critical micronutrient for plant growth and development. It is involved in numerous physiological and biochemical processes, including chlorophyll synthesis, photosynthesis, electron transport, and enzymatic activity [4]. Iron deficiency is a widespread constraint in pigeonpea cultivation, particularly in calcareous and marginal soils, which can reduce germination rates by 15–30% and lower overall yields by 20–40% [1, 5]. Insufficient iron during early seedling development can lead to chlorosis, stunted growth, and weak root systems, limiting the plant’s ability to access water and nutrients. Such deficiencies are especially problematic in semi-arid and tropical regions where pigeonpea is predominantly grown, often on nutrient-poor soils. Addressing iron limitation at the seed stage through targeted interventions, such as seed priming with iron-enriched formulations, is therefore critical for ensuring uniform crop establishment and achieving optimal yield potential.

Seed priming is a pre-sowing treatment that improves seed performance by initiating the early stages of germination without allowing radicle emergence. This technique enhances germination and seedling vigor by improving metabolic activity and preparing seeds for optimal growth conditions. Various seed priming methods, including hydropriming, osmopriming, biopriming, and nutrient priming, have been widely used to improve germination, early seedling development, and dormancy breaking in various plant species [6]. These methods enhance the physiological and biochemical processes within the seed, making them better equipped to handle abiotic stresses and ensuring uniform crop establishment. For example, osmopriming involves the use of osmotic solutions to regulate water uptake, while biopriming incorporates beneficial microorganisms to improve seedling vigor and resilience [7, 8].

Green-synthesized nano-iron provides a sustainable and effective solution to combat iron deficiency in plants. These nanoparticles offer enhanced solubility, bioavailability, and targeted delivery of iron, thereby addressing the challenges associated with conventional iron fertilizers. Their unique properties, such as high surface area, small particle size, and reactive surfaces, allow for more efficient nutrient uptake by plants [9, 10]. Additionally, the controlled release behavior of these nanoparticles ensures a steady supply of iron during critical growth phases, making them particularly beneficial for improving seed germination and early plant development [11]. Additionally, the presence of bioactive compounds from the green synthesis process may further enhance plant growth and reduce oxidative stress.

Although the benefits of nanotechnology in agriculture have been widely explored, studies specifically examining the use of green-synthesized iron nanoparticles as a seed priming agent in pigeonpea are extremely limited. To the best of our knowledge, no prior research has evaluated the combined advantages of (i) eco-friendly green synthesis of nano-iron, (ii) its application through seed priming, and (iii) its direct effects on improving the germination physiology and early seedling vigor of pigeonpea. This study uniquely integrates green nanotechnology with seed priming—a low-cost, farmer-friendly technique—to develop a sustainable and efficient strategy for addressing iron deficiency at the earliest growth stages. The work therefore fills a significant research gap and offers a novel approach to enhancing pigeonpea establishment and productivity under resource-limited and iron-deficient conditions. Accordingly, the present study aimed to evaluate the effectiveness of green-synthesized iron nanoparticles as a seed-priming agent to improve germination and early seedling vigor in pigeonpea.

## Material and method

### Initial screening of leaf sample for synthesis of iron nano particles

**Fig. 1:**
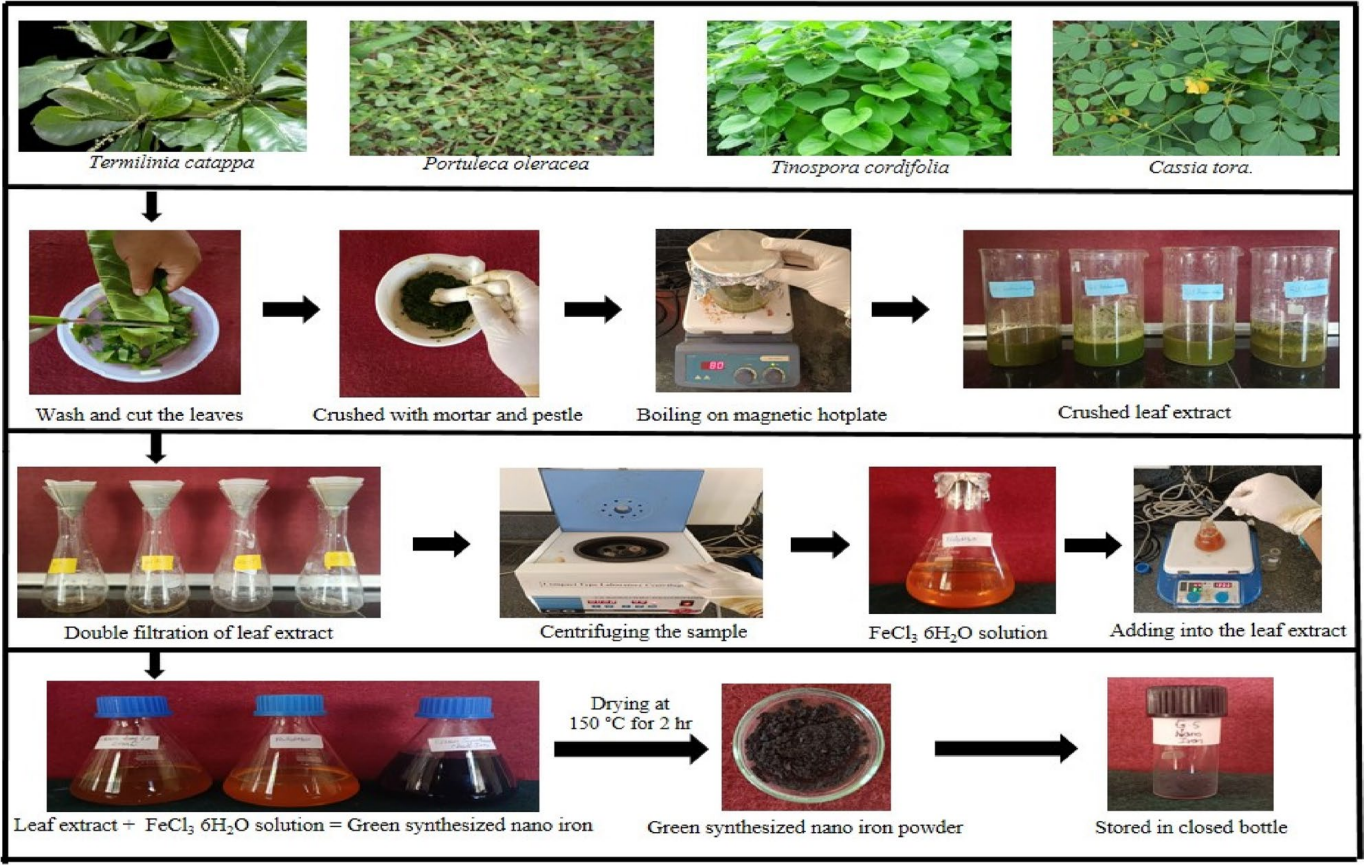
Schematic representation of synthesis of iron nanoparticles from different leaf extract

Various plant extracts such as *Terminalia catappa*, *Portulaca oleracea*, *Tinospora cordifolia*, and *Cassia tora* were employed in the preliminary screening of selected plant leaf samples to identify the most suitable extract. The leaf samples were systematically collected from the Field Unit of the University of Agricultural Sciences, Raichur, in adherence to institutional protocols and standard field collection practices. The leaf extract was employed as a capping and reducing agent. When leaf extracts were combined with FeCl_3_ solution, Fe^+ 3^ ions were converted into Fe^0^ nanoparticles [4, 12]. The synthesis was performed under ambient indoor light, as the rapid reduction and chelation of Fe³⁺ by the polyphenol-rich extract prevented any light-induced alteration in FeCl₃. Visible confirmation of synthesized nano iron was done with change in colour of leaf extract (Fig. 1). Further confirmation of size was done through particle size analysis. The finest leaf extract sample for synthesis of nano iron came from *Terminalia catappa* which is rich in iron.

### Synthesising the nano iron particles using the leaf extract

Fresh leaves of *Terminalia catappa* were collected from the field unit at UAS, Raichur campus. The leaves were thoroughly washed under running tap water to remove debris and dust, followed by rinsing with distilled water, and then dried at room temperature. The dried leaves were cut into small pieces using an ethanol-sterilized knife, crushed with a sterilized mortar and pestle, and dispensed in 200 ml of distilled water. The crushed sample was transferred to a 500 ml beaker and boiled at 70 °C for 30 min. The aqueous extract of the leaves was separated through double filtration using Whatman No.1 filter paper and centrifuged at 1000 rpm for 5 min to remove biomaterials. The filtrate was collected and stored at room temperature for further use.

For the synthesis of iron oxide nanoparticles, a 0.01 M aqueous solution of FeCl₃·6 H₂O was prepared by dissolving 0.270 g of the compound in 100 ml of water. The aqueous solution was then mixed with 50 ml of the green leaf extract in different proportions (1:1, 2:1, and 4:1) with constant stirring using a magnetic stirrer for 30 min. During the stirring process, the solution turned black, indicating nanoparticle formation [4]. The best ratio for synthesis was standardized based on the results. After stirring, the solution was left undisturbed for 3 h, and the supernatant was collected and centrifuged at 5000 rpm for 30 min. The resulting nanoparticles were dried in a hot air oven at 150 °C for 2 h and stored in a sealed container for further analysis. The pH and characterization of the synthesized iron oxide nanoparticles were then performed.

### Characterization of synthesised nano iron particles

After synthesizing the nano iron particles, a series of instrumental analyses were conducted to characterize their properties. Preliminary analysis was performed using UV-vis spectrophotometry, operating within a calibration range of 215 to 650 nm [13, 14]. For this, a diluted aqueous dispersion of the nanoparticles was prepared and transferred into a quartz cuvette for spectral scanning. The particle size distribution was measured using the Zetasizer tool, which applies the dynamic light scattering (DLS) principle [3]. The sample was sonicated to avoid agglomeration and filtered to remove dust before analysis. Detailed surface morphology and nanoscale size were examined using Scanning Electron Microscopy (SEM) and Transmission Electron Microscopy (TEM), with TEM providing higher resolution. For SEM analysis, a small amount of dried nanoparticle powder was mounted on carbon tape over an aluminum stub and sputter-coated with gold to ensure conductivity. For TEM, a drop of well-dispersed nanoparticle suspension in ethanol was placed on a copper grid with a carbon film and dried under infrared light. Energy Dispersive X-ray Spectroscopy (EDS), performed alongside SEM, confirmed the elemental composition of the nanoparticles [15]. Fourier Transform Infrared Spectroscopy (FTIR) was used to identify the functional groups and biomolecules involved in nanoparticle stabilization. The FTIR sample was prepared by mixing dried nanoparticle powder with spectroscopic grade KBr, pelletizing it under pressure, and then scanning in the range of 4000–400 cm⁻¹. These comprehensive analyses provided critical insights into the physical, chemical, and structural properties of the synthesized nanoparticles [16].

### Experiment on seed germination

The experiment was conducted in a factorial completely randomized block design (FCRD) in a laboratory setting with four replications with 100 seeds in each repetition. Germination percentage was recorded on the 4th day and other germination parameters was recorded 6 days after sowing [17]. Seedling vigor index was calculated following the ISTA procedure (Anon., 2011). Factor I examined forms of fertilizer: green-synthesized (GS) nano iron (P_1_) and commercial product (CP) nano iron (P_2_). Factor II evaluated fertilizer concentrations: 25 ppm (F_1_), 50 ppm (F_2_), 75 ppm (F_3_), 100 ppm (F_4_), and 125 ppm (F_5_). The interaction between the type of nano-iron fertilizer (GS vs. CP) and its concentration (25 ppm to 125 ppm) was examined through various treatments. For example, T_1_ to T_5_ used GS nano-iron at increasing concentrations (25 ppm to 125 ppm), while T_6_ to T_10_ applied CP nano-iron at the same concentrations. Control treatments included seed priming with distilled water (C_1_) and FeSO₄ at 0.1% (C_2_).

### Observations on germination parameters and seed vigour of pigeonpea

Germination parameters in pigeonpea was assessed after 4 and 6 days of incubation by measuring shoot length, root length, germination percentage, and seedling vigor index [18]. Germination percentage was based on normal seedlings, while abnormal seedlings and dead seeds were recorded as percentages [19]. Shoot and root lengths were measured, and seedling dry weight was determined by drying normal seedlings at 70 °C for 24 h. Mean germination time (MGT), speed of germination, mean daily germination (MDG), peak value (PV), and germination value (GV) were calculated using standard formulas [20, 21].

#### Germination percentage (%)

The number of normal seedlings in each replication was taken at the final count. Germination was calculated and expressed in percentage as mentioned below

$$\:\mathrm{G}\mathrm{e}\mathrm{r}\mathrm{m}\mathrm{i}\mathrm{n}\mathrm{a}\mathrm{t}\mathrm{i}\mathrm{o}\mathrm{n}\:\left(\mathrm{\%}\right)\:=\frac{\:\mathrm{N}\mathrm{o}.\:\mathrm{o}\mathrm{f}\:\mathrm{n}\mathrm{o}\mathrm{r}\mathrm{m}\mathrm{a}\mathrm{l}\:\mathrm{s}\mathrm{e}\mathrm{e}\mathrm{d}\mathrm{n}\mathrm{g}\mathrm{s}}{\mathrm{T}\mathrm{o}\mathrm{t}\mathrm{a}\mathrm{l}\:\mathrm{n}\mathrm{u}\mathrm{m}\mathrm{b}\mathrm{e}\mathrm{r}\:\mathrm{o}\mathrm{f}\:\mathrm{s}\mathrm{e}\mathrm{e}\mathrm{d}\mathrm{s}}\times\:100$$

#### Abnormal seedlings (%)

From the germination test, those seedlings which did not show the capacity for continued development into normal plants under favorable conditions of water supply, temperature and light were counted and expressed in percentage.

$$\:\mathrm{A}\mathrm{b}\mathrm{n}\mathrm{o}\mathrm{r}\mathrm{m}\mathrm{a}\mathrm{l}\:\mathrm{s}\mathrm{e}\mathrm{e}\mathrm{d}\mathrm{l}\mathrm{i}\mathrm{n}\mathrm{g}\:\left(\mathrm{\%}\right)\:=\frac{\:\mathrm{N}\mathrm{o}.\:\mathrm{o}\mathrm{f}\:\mathrm{a}\mathrm{b}\mathrm{n}\mathrm{o}\mathrm{r}\mathrm{m}\mathrm{a}\mathrm{l}\:\mathrm{s}\mathrm{e}\mathrm{e}\mathrm{d}\mathrm{l}\mathrm{i}\mathrm{n}\mathrm{g}\mathrm{s}}{\mathrm{T}\mathrm{o}\mathrm{t}\mathrm{a}\mathrm{l}\:\mathrm{n}\mathrm{u}\mathrm{m}\mathrm{b}\mathrm{e}\mathrm{r}\:\mathrm{o}\mathrm{f}\:\mathrm{s}\mathrm{e}\mathrm{e}\mathrm{d}\mathrm{s}}\times\:100$$

#### Dead seeds (%)

From the germination test, those seeds which at the end of test period, were neither hard nor fresh and have not produced seedlings were counted and expressed in percentage.

$$\:\mathrm{D}\mathrm{e}\mathrm{a}\mathrm{d}\:\mathrm{s}\mathrm{e}\mathrm{e}\mathrm{d}\mathrm{s}\:\left(\mathrm{\%}\right)\:=\frac{\mathrm{N}\mathrm{o}.\:\mathrm{o}\mathrm{f}\:\mathrm{d}\mathrm{e}\mathrm{a}\mathrm{d}\:\mathrm{s}\mathrm{e}\mathrm{e}\mathrm{d}\mathrm{s}}{\mathrm{T}\mathrm{o}\mathrm{t}\mathrm{a}\mathrm{l}\:\mathrm{n}\mathrm{u}\mathrm{m}\mathrm{b}\mathrm{e}\mathrm{r}\:\mathrm{o}\mathrm{f}\:\mathrm{s}\mathrm{e}\mathrm{e}\mathrm{d}\mathrm{s}}\times\:100$$

#### Shoot length (cm)

The shoot length of germinated seeds was measured from the tip of shoot to the hypocotyl point and the mean length was calculated and expressed in centimeters.

#### Root length (cm)

The root length of germinated seeds was measured from tip of root to the hypocotyl point and the mean length was calculated and expressed in centimeters.

#### Seedling dry weight (mg)

Ten normal seedlings used for measuring shoot and root length were dried in a hot-air oven at 70˚C for 24 h. After cooling in a desiccator for 20 min, their weight was recorded using an electronic balance, and the average weight was expressed in milligrams.

#### Mean germination time (MGT)

The mean number of days required for total germination for a particular treatment was worked by the following formula described by Maguire [20].

$$\mathrm{MGT}=\frac{{\mathrm N}_1{\mathrm D}_1\;+\;{\mathrm N}_2{\mathrm D}_2\;+\;...+\;{\mathrm N}_{\mathrm k}{\mathrm D}_{\mathrm k}}{\mathrm{Total}\;\mathrm{number}\;\mathrm{of}\;\mathrm{seeds}}\times100$$

Where,

N = Number of germinated seeds.

D = Number of days.

### Speed of germination

Seeds were germinated on paper with four replicates of 100 seeds each. Daily germination was recorded until the final count, and the speed of germination was calculated using Maguire’s formula [20].

$$\:\mathrm{S}\mathrm{p}\mathrm{e}\mathrm{e}\mathrm{d}\:\mathrm{o}\mathrm{f}\:\mathrm{g}\mathrm{e}\mathrm{r}\mathrm{m}\mathrm{i}\mathrm{n}\mathrm{a}\mathrm{t}\mathrm{i}\mathrm{o}\mathrm{n}\:=\frac{\mathrm{X}1}{\mathrm{Y}1}+\frac{\mathrm{X}2-\mathrm{X}1}{\mathrm{Y}2}+\dots\:+\frac{\mathrm{X}\mathrm{n}-(\mathrm{X}\mathrm{n}-1)}{\mathrm{Y}\mathrm{n}}$$

Where,

Xn = Number of seeds germinated at n^th^ count.

Yn = Number of days from sowing to n^th^ count.

#### Mean daily germination (MDG)

The final germination percentage at the end of the test divided by the number of days was expressed as mean daily germination.

$$\:\mathrm{M}\mathrm{D}\mathrm{G}\:=\frac{\mathrm{T}\mathrm{o}\mathrm{t}\mathrm{a}\mathrm{l}\:\mathrm{n}\mathrm{u}\mathrm{m}\mathrm{b}\mathrm{e}\mathrm{r}\:\mathrm{o}\mathrm{f}\:\mathrm{g}\mathrm{e}\mathrm{r}\mathrm{m}\mathrm{i}\mathrm{n}\mathrm{a}\mathrm{t}\mathrm{e}\mathrm{d}\:\mathrm{s}\mathrm{e}\mathrm{e}\mathrm{d}\mathrm{s}}{\mathrm{T}\mathrm{o}\mathrm{t}\mathrm{a}\mathrm{l}\:\mathrm{n}\mathrm{u}\mathrm{m}\mathrm{b}\mathrm{e}\mathrm{r}\:\mathrm{o}\mathrm{f}\:\mathrm{d}\mathrm{a}\mathrm{y}\mathrm{s}}\times\:100$$

#### Peak value (pv)

Peak value as the quotient of final germination percentage and number of days required to reach the peak value.

$$\:\mathrm{p}\mathrm{v}\:=\frac{\mathrm{F}\mathrm{i}\mathrm{n}\mathrm{a}\mathrm{l}\:\mathrm{g}\mathrm{e}\mathrm{r}\mathrm{m}\mathrm{i}\mathrm{n}\mathrm{a}\mathrm{t}\mathrm{i}\mathrm{o}\mathrm{n}\:\mathrm{p}\mathrm{e}\mathrm{r}\mathrm{c}\mathrm{e}\mathrm{n}\mathrm{t}\mathrm{a}\mathrm{g}\mathrm{e}}{\mathrm{N}\mathrm{u}\mathrm{m}\mathrm{b}\mathrm{e}\mathrm{r}\:\mathrm{o}\mathrm{f}\:\mathrm{d}\mathrm{a}\mathrm{y}\mathrm{s}\:\mathrm{r}\mathrm{e}\mathrm{q}\mathrm{u}\mathrm{i}\mathrm{r}\mathrm{e}\mathrm{d}\:\mathrm{t}\mathrm{o}\:\mathrm{r}\mathrm{e}\mathrm{a}\mathrm{c}\mathrm{h}\:\mathrm{p}\mathrm{e}\mathrm{a}\mathrm{k}\:\mathrm{v}\mathrm{a}\mathrm{l}\mathrm{u}\mathrm{e}}\times\:100$$

#### Germination value (GV)

Germination value of a seed lot as the product of peak value and the mean daily germination.

$$\mathrm{GV}=\mathrm{Peak}\;\mathrm{value}\;\times\;\mathrm{Mean}\;\mathrm{daily}\;\mathrm{germination}$$

### Dehydrogenase enzyme activity

Twenty-five representative seeds from each treatment were soaked overnight at room temperature, then steeped in a 0.25% solution of 2,3,5-triphenyl tetrazolium chloride in the dark at 40 °C for two hours. After staining, the seeds were washed, soaked in 10 ml of methyl cellosolve overnight to extract the red formazan, and the intensity of red color was measured using an ELICO UV-VIS spectrophotometer (model SC-159) at 470 nm. Methyl cellosolve served as the blank, and the OD value was recorded as dehydrogenase activity [22].

### Alpha amylase activity (mm)

Alpha amylase activity was determined using the method of Simpson and Naylor [23]. A paste was prepared by mixing 2 g of agar shreds and 1 g of potato starch in water, then making up the volume to 100 ml with distilled water. This mixture was boiled, poured into sterilized petri dishes, and allowed to form a gel. Pre-soaked (8 h) and halved seeds, with the cotyledon and embryo intact, were placed on the gel. The petri dishes were kept in the dark at 30 °C for 24 h. Afterward, potassium iodide solution (0.44 g iodine + 20.008 g potassium iodide in 500 ml distilled water) was applied, and the excess was drained. The diameter of the clear halo around the seed was measured and reported as α-amylase activity.

### Statistical analysis

The seed germination experiments was executed under laboratory condition in a factorial completely randomized design (FCRD) with four replications. The mean values were presented with standard deviation in the figures. The test was performed using SPSS software developed by SPSS Inc, IBM.

## Results and discussions

### Visible confirmation of synthesis of iron nano particles

The synthesis of iron nanoparticles was done using the leaf extract of *Terminalia catappa*,* Portuleca oleracea*,* Tinospora cordifolia and Cassia tora*. The leaves extract was used as reducing as well as capping agent. Fe^+ 3^ ions were reduced into Fe^0^ nanoparticles when leaves extracts was mixed with FeCl_3_6H_2_O solution [24]. The leaves extract when mixed with bright yellowish colour of ferric chloride solution and it was changed to black colour along with reduction in pH from 6.83 to 2.97 (*Terminalia catappa*), 6.44 to 2.87 (*Portuleca oleracea*), 6.88 to 3.94 (*Tinospora cordifolia*) and 7.62 to 2.85 (*Cassia tora*), The intensity of colour increased steadily up to 3 h and it turned black colour within 24 h indicated the synthesis of nano iron particles (presented in Table 1). After 24 h there was no significant colour change, which was evidence for the completion of reduction reaction. Among these selected species, *Terminalia catappa* contains higher amount of phenolics and considered as good reducing and stabilising agent. If leaf extractant contains higher amount of phenolics then it reduces the particles to nano size. Similar results reported in several reports [14, 25].

**Table 1:** Colour change and reduction in pH during synthesis of iron nano particles

Leaf extract	Initial colour	Final colour	Initial pH	Final pH
*Terminalia catappa*,	Light green	Black	6.83	2.97
*Portuleca oleracea*,	Light Brown	Black	6.44	2.87
*Tinospora cordifolia*	Brown	Black	6.97	3.94
*Cassia tora*	Light orange	Brown	7.72	2.85

### Characterization of synthesized nano iron particles

#### Dynamic light scattering (DLS) analysis

The hydrodynamic size and polydispersity index (PDI) of the green-synthesized iron nanoparticles were measured using a Malvern Zetasizer Nano ZS (Model Nano Z383) based on quasi-elastic light scattering. Prior to analysis, the colloidal suspension was diluted 1:10 with ultrapure water to minimize multiple scattering effects. The refractive index and viscosity inputs were set according to standard values for iron oxide dispersions (RI: 2.42) and aqueous media at 25 °C, respectively. Each sample was analyzed in triplicate, with three measurement runs recorded per replicate to ensure accuracy and reproducibility. The size distribution results indicated that 95% of the particles measured 82.74 nm (SD 36.2 nm), while 5.3% measured 16.66 nm (SD 4.3 nm). The average hydrodynamic sizes obtained for nanoparticles synthesized using different plant extracts were 72.72 nm (*Terminalia catappa*), 73.03 nm (*Portulaca oleracea*), 433.31 nm (*Tinospora cordifolia*), and 233.12 nm (*Cassia tora*), as presented in Table 2. These results demonstrate a relatively homogeneous distribution of particles and align with the findings of earlier studies [18].

### Zeta potential analysis

Zeta potential of green synthesized nano iron refers to the electrokinetic potential difference between the surface of the nanoparticles and the surrounding dispersing medium. In this case zeta potential of synthesized nano iron was -−21.2 mV (*Terminalia catappa*), −18.9 mV (*Portuleca oleracea*), −17.4 mV (*Tinospora cordifolia*) and − 14.5 mV (*Cassia tora*) (presented in Table 2), a negative zeta potential is often observed due to the presence of surface oxide layers, which contribute to repulsive forces between particles, preventing agglomeration and enhancing stability [26]. Biomolecules present in plant extracts and other biological reducing agents adsorb onto the surface of green-synthesized nanoparticles, forming a biomolecular “halo” or corona that stabilizes the particles, prevents aggregation, and modulates their reactivity and interaction with biological systems [27, 28].

### Spectrophotometric analysis of nanoparticles

UV-Visible (UV-Vis) spectrophotometry enables the analysis of the absorption spectra of nanoparticles synthesized through green methods. This technique also facilitates quantitative analysis, correlating absorbance intensity with nanoparticle concentration according to the Beer-Lambert law. Results indicated that the main absorbance peaks of iron nanoparticles were observed in the range of 230 to 324 nm, with the peak at 239 nm (*Terminalia catappa*), 324 nm (*Portuleca oleracea*), 288 nm (*Tinospora cordifolia*) and 233 nm (*Cassia tora*) indicating successful formation of iron nanoparticles (presented in Table 2). These peaks are likely due to the SPR effect (surface plasmon resonance), where the nanoparticles exhibit specific optical features depending on their size, shape, and the specific biomolecules involved in the reduction of iron ions. The variation in peak positions across different plant extracts suggests that the phytochemicals in each extract influence the formation of the nanoparticles differently, potentially affecting their size, shape, and stability [3].

**Table 2:** Zetasizer, zeta potential and UV-Vis spectral analysis values of green synthesized iron nanoparticles

Selected plant sample for nano iron synthesis	Particle size in nanometer (nm)	Zeta potential in millivolt (mV)	Maximum deflection (nm)
*Terminalia catappa*,	72.72	−21.2	239
*Portuleca oleracea*,	73.03	−18.9	324
*Tinospora cordifolia*	433.31	−17.4	288
*Cassia tora*	233.12	−14.5	233

### Scanning electron microscope with EDAX

**Fig. 2:**
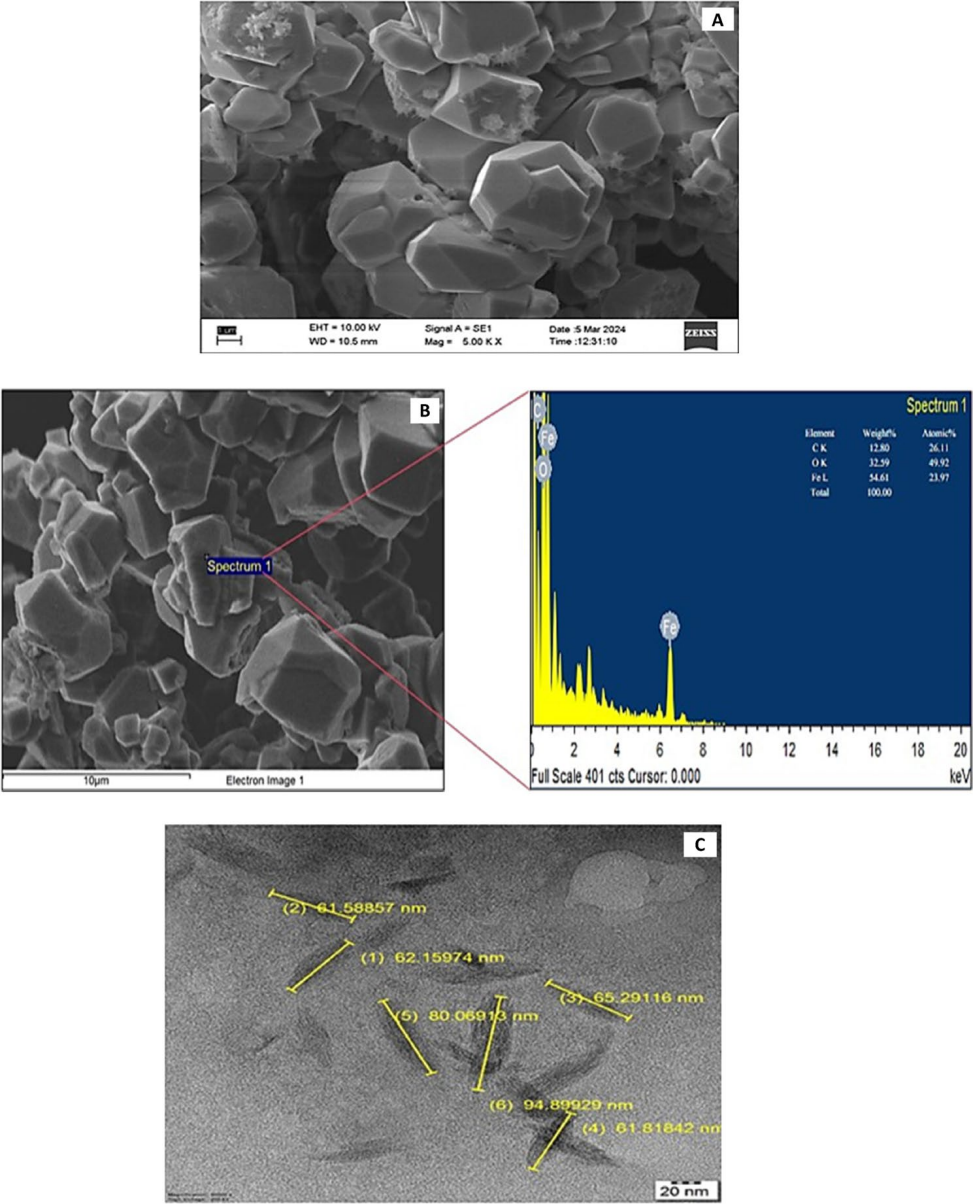
SEM (Scanning electron microscope) characterization result (**A**), EDAX characterization result (**B**) and TEM (Transmission Electron Microscope) characterization result (**C**)

SEM was utilized to determine the size of the green synthesized nano iron particles. SEM operates on the principle of magnifying the target object, offering magnifications of up to 1–2 million times. This approach allows for precise characterization of particle size. The nano iron particle sample exhibited sizes less than 100 nm and demonstrated homogeneous dispersion. During SEM measurements, larger particles were observed, likely due to the aggregation of smaller ones [29]. The shape of the nanoparticles, characterized by their special nature, appeared to be interconnected (Fig. 2, A). EDAX spectra analysis of the selected regions indicated that the green synthesized nano iron particles comprised predominantly of Fe (54.61 wt %), C (17.73 wt %) and O (32.39%). The result of EDAX gives a clear idea about the elements present in the biosynthesized nanoparticles (Fig. 2, B). The presence of carbon and oxygen in addition to iron is a significant observation, as these elements may stem from the biomolecules that act as reducing agents or stabilizers in the green synthesis process. These biomolecules, such as polyphenols or proteins, can cap the surface of the nanoparticles, preventing further aggregation and enhancing stability [30, 31].

### Transmission electron microscope (TEM) studies

TEM imaging revealed distinct differences in size, pores, and morphology between commercially available and green-synthesized nano iron particles. With magnifications of up to 50 million times, Selective Area Electron Diffraction (SAED) patterns confirmed the nanocrystalline nature of the particles. The results showcased that green-synthesized nano iron particles exhibited a spherical elongated morphology with porosity, with pore sizes ranging from 8 to 12 nm and sizes ranging from 60 to 90 nm (Fig. 2, C). Considering the ionic radius of iron ions at approximately 0.74 Å (1 Å = 0.1 nm), this evidence suggests a significant change in particle morphology during the conversion into nano form, driven by the interaction of plant-based reducing and capping agents with metal precursors. These biomolecules influence nucleation and growth rates, contributing to the formation of porous and highly reactive structures. The porous nanoparticles, such as those observed in the green synthesis, exhibit enhanced surface-to-volume ratios, which improve their reactivity and penetration into plant tissues. This enhanced accessibility to active sites facilitates potential applications in agriculture, including nutrient delivery and pest control, as well as in environmental remediation, where their high reactivity can support pollutant degradation [32].

### Fourier transform infrared spectroscopy (FT-IR)

**Fig. 3:**
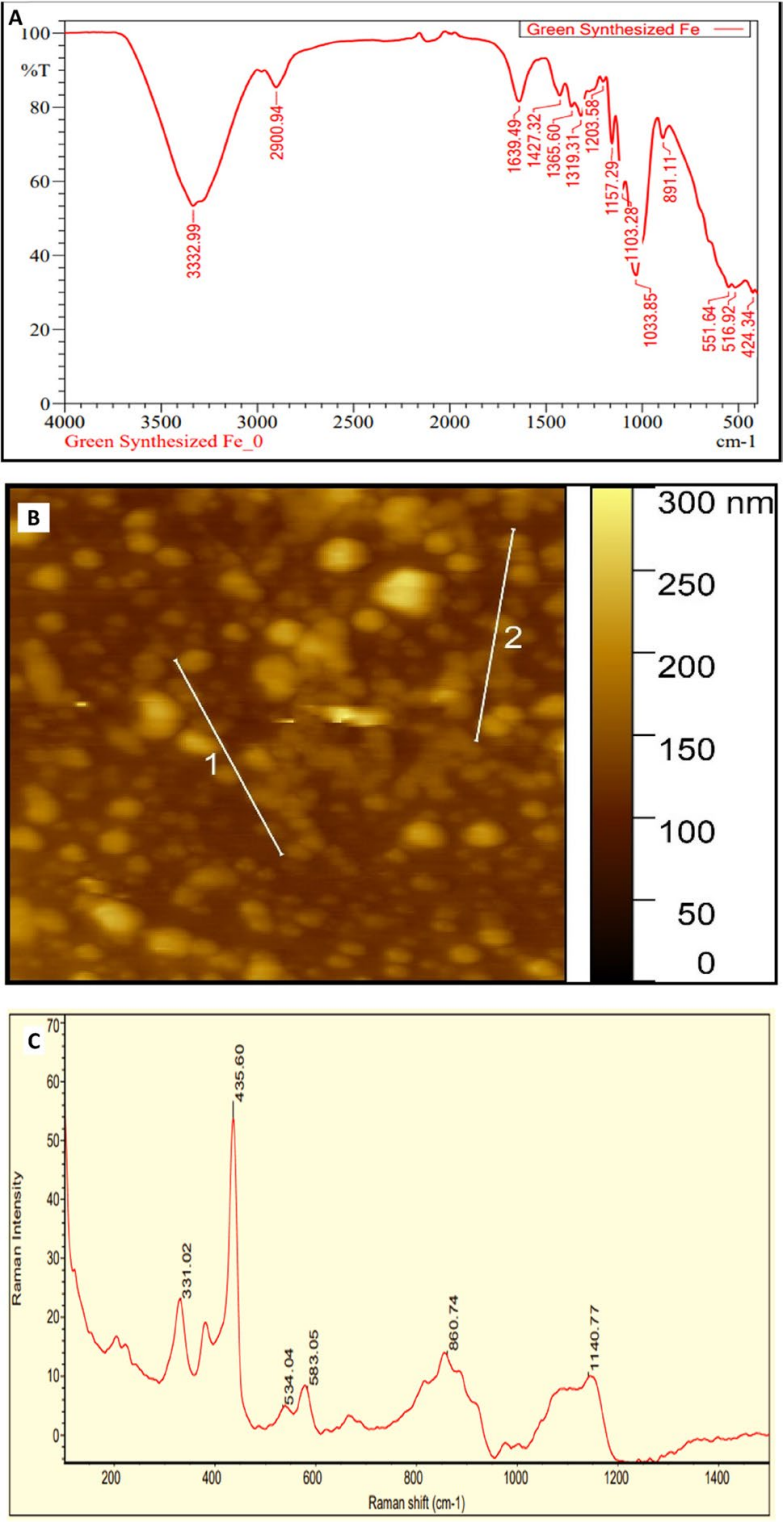
FTIR characterization result (**A**), AFM characterization result (**B**) and Raman spectroscopy characterization result (**C**)

Fourier transform infrared spectroscopy (FT-IR) analysis of green synthesized nano iron revealed distinctive spectra indicative of their chemical composition and functional groups. In the case of nano iron, FT-IR spectra exhibited characteristic peaks corresponding to iron oxide (Fe-O) bonds, confirming the presence of iron nanoparticles. Additionally, peaks associated with organic molecules from the plant extract used in the synthesis process were observed, suggesting their involvement in nanoparticle stabilization. The broad peak at 3332 cm ^− 1^ is indicative of the presence of OH groups. The stretching vibration of the carbonyl group is observed at 1639 cm^− 1^, while the peak at around 1033 cm^− 1^ is attributed to C–O bonds. Peaks corresponding to aliphatic C–H bonds appear around 2900 cm^− 1^. These peaks suggest that the nanoparticles were functionalized with organic compounds, likely from the *Terminalia catappa*, extract used in the synthesis process. Functionalization of nanostructures with hydrophilic groups such as hydroxyl, carbonyl, and C–O increases their interactions with water molecules, resulting in more stable colloids. Iron nanoparticles typically exhibit intense and sharp peaks at about 551 cm^− 1^ and 424 cm^− 1^ due to Fe–O bonds. However, these peaks were not observed in the FTIR spectrum of leaf etract of *Terminalia catappa*, indicating that they were zero-valent iron nanoparticles (Fig. 3A). FT-IR spectra of leaf extract of *Terminalia catappa* summarized presence of a broad band around 3350 cm^− 1^. The peak values at 1640.0 cm^− 1^ indicative of the presence of aliphatic compounds in leaf extract of *Terminalia catappa*. Zero-valent iron nanoparticles exhibit characteristics of both iron oxide/hydroxide and zero-valent iron. Similar spectra have been reported by [9, 33].

### Atomic force microscope (AFM)

The AFM analysis of green-synthesized nano iron particles revealed intriguing findings. The 3D AFM image displayed a distinctive folded, mountain-like structure, indicative of a complex and irregular morphology. This structure could be attributed to metal bindings or the deposition of oxide layers during synthesis. The average length of these structures ranged from 0 to 300 nm, reflecting a diverse range of particle sizes. This diversity in size and morphology is characteristic of green synthesis methods, where various phytochemicals influence the growth and aggregation of nanoparticles. The lines marked as “1” and “2” represent cross-sectional regions used for height analysis, highlighting the heterogeneous surface morphology (Fig. 3B). The nanoscale dimensions and observed particle aggregation can be linked to the high surface energy and reactivity of the particles. This surface roughness and particle uniformity are critical for applications such as catalysis, adsorption, and nutrient delivery, as they enhance surface area and reactivity, confirming the nanoparticles’ synthesis and functional potential. Similar findings have been reported in previous studies [34, 35]. The height profile analysis indicated an average particle diameter of approximately 65 nm, with a size distribution ranging from 53 to 290 nm.

### Raman spectroscopy

The Raman spectrum reveals prominent peaks at 435.60 cm⁻^1^ and 331.02 cm⁻^1^, which are characteristic of lattice vibrations or metal-oxygen bonds, often indicative of iron oxide or similar crystalline nanomaterials. Peaks at 524.04 cm⁻^1^ and 583.05 cm⁻^1^ likely correspond to specific stretching vibrations associated with the material’s bonding framework, such as Fe-O interactions. The broad feature at 860.74 cm⁻^1^ may reflect secondary vibrational modes or structural heterogeneity, while the peak at 1140.77 cm⁻^1^ could indicate functional groups or surface modifications due to synthesis conditions. These features suggest a mix of crystalline and amorphous phases, with the strong 435.60 cm⁻^1^ peak denoting the primary crystalline phase (Fig. 3C). Similar observations were reported by [33, 36] in studies of resonant Raman scattering in iron oxide systems, where lattice layer effects and resonance conditions amplified Fe-O vibrational signatures. This dual-phase characteristic enhances the material’s functional properties, particularly in applications requiring both stability (from the crystalline phase) and adaptability (from the amorphous phase).

### Investigating the effects nano iron on germination parameters of pigeonpea

**Fig. 4:**
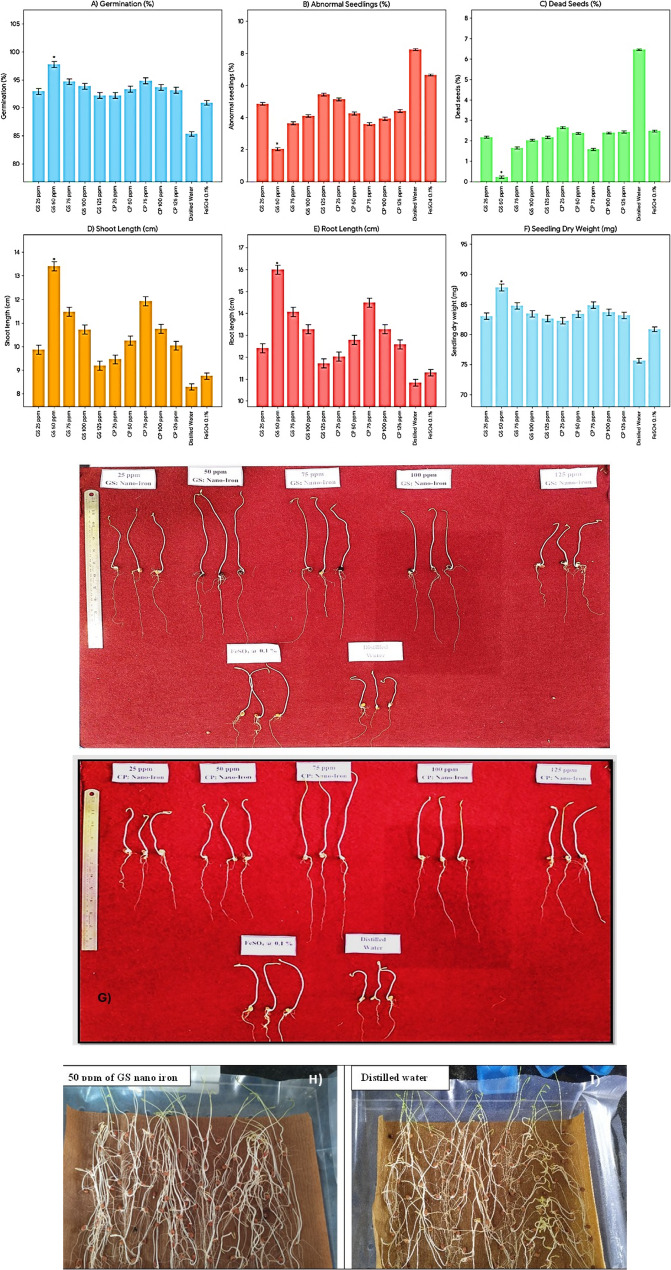
Bar graphs showing the effects of green-synthesized and commercially produced iron nanoparticles on pigeonpea germination and early seedling growth. Images (**A**–**F**) represent germination percentage, abnormal seedlings (%), dead seeds (%), shoot length (cm), root length (cm), and seedling dry weight (mg), respectively. Error bars indicate standard deviations (*n* = 3). Images (**G**–**I**) visually depict the germination response of pigeonpea seeds under different nanoparticle treatments

The study results showed that green synthesized (GS) nano-iron consistently outperformed the commercial product (CP) nano-iron in various germination and seedling parameters (Fig. 4). GS nano-iron achieved higher germination percentages (94.30% vs. 93.44%) and lower abnormal seedling percentages (4.01% vs. 4.26%). It also had fewer dead seeds (1.65% vs. 2.28%) and better shoot length (10.93 cm vs. 10.48 cm), root length (13.50 cm vs. 13.04 cm), and seedling dry weight (84.35 mg vs. 83.49 mg). Among the fertilizer concentrations, 50 ppm proved to be the most effective, with the highest germination percentage (95.55%), lowest abnormal seedlings (3.14%), and dead seeds (1.29%). This concentration also resulted in the best shoot length (11.83 cm), root length (14.40 cm), and seedling dry weight (85.59 mg) (presented in Fig. 4). The interaction data revealed that seed priming with GS nano-iron at 50 ppm produced the best overall results, with a germination percentage of 97.76%, the lowest abnormal seedlings (2.03%), and dead seeds (0.22%). It also resulted in the highest shoot length (13.41 cm), root length (16.00 cm), and seedling dry weight (87.81 mg). While other combinations, such as GS nano-iron at 75 ppm and 100 ppm, also performed well, the combination of GS nano-iron at 50 ppm was the most effective for enhancing seedling growth. Control treatments, particularly distilled water priming, showed significantly lower performance in all parameters, highlighting the positive impact of nano-iron priming. Nano iron enhances enzymatic activity and nutrient uptake, promoting metabolic processes critical for seedling growth [37, 38]. The superior performance of green-synthesized nano-iron may be attributed to the presence of bioactive phytochemicals on the nanoparticle surface, which enhance stability, uptake efficiency, and physiological responsiveness compared to commercially produced nano-iron [13, 39]. The biological effects of nanoparticles, including both efficacy and phytotoxicity, are strongly influenced by their physicochemical characteristics such as size, shape, and concentration, as well as by the plant species exposed to them. Smaller nanoparticles typically exhibit higher reactivity and bioavailability, which can enhance nutrient uptake but may also increase the risk of phytotoxicity at higher concentrations. Similarly, the shape of nanoparticles can affect their interaction with plant tissues, influencing absorption, translocation, and cellular responses. Plant species also play a critical role, as their sensitivity to nanoparticles varies depending on physiology and metabolic capacity. Therefore, careful optimization of nanoparticle characteristics and application rates is essential to maximize beneficial effects while minimizing potential toxicity in crops [10, 27].

### Investigating the effects nano iron on seed vigour potential of pigeonpea

**Fig. 5:**
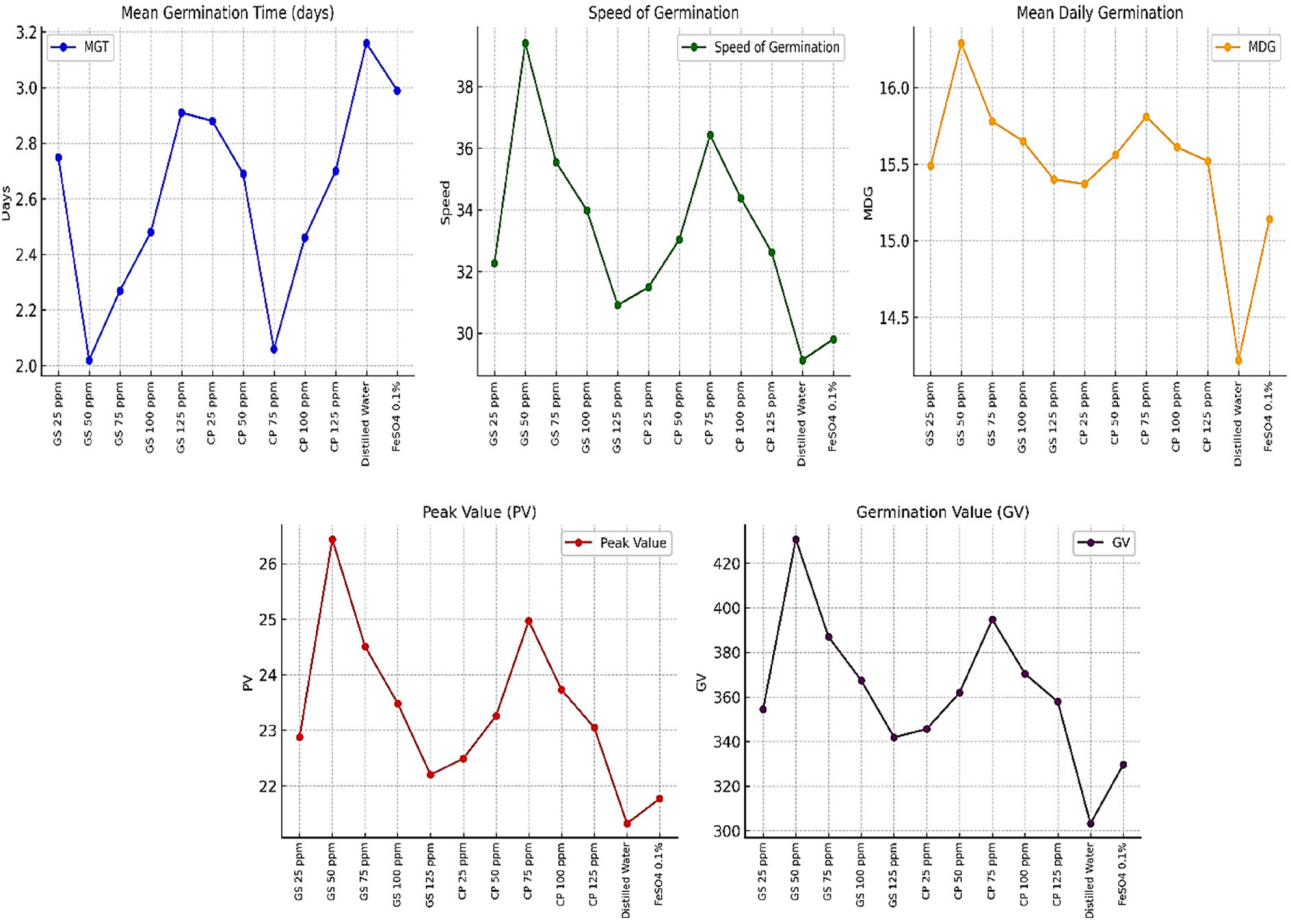
Effect of green synthesized and commercial product nano iron particles on seed vigour potential of pigeonpea

The study revealed that green synthesized (GS) nano-iron consistently outperformed the commercial product (CP) nano-iron across most parameters. GS nano-iron exhibited faster germination (mean germination time: 2.49 days vs. 2.56 days), higher speed of germination (34.43 vs. 33.59), and superior germination value (376.28 vs. 366.12). Among fertilizer concentrations, 50 ppm was the most effective, recording the shortest germination time (2.17 days), the highest speed of germination (36.23), peak value (24.85), and germination value (396.32). Interaction data highlighted that seed priming with green synthesized nano-iron at 50 ppm achieved the best results, with a mean daily germination of 16.29, the highest peak value of 26.44, and germination value of 430.72 (presented in Fig. 5). Other effective combinations included green synthesized nano-iron at 75 ppm and 100 ppm, both showing commendable germination parameters. Similarly, among treatments with commercial product nano-iron, 75 ppm performed relatively well. However, higher concentrations, such as 125 ppm for both forms, showed reduced effectiveness, suggesting possible inhibition at elevated levels [40, 41] Control treatments, especially distilled water priming, demonstrated significantly lower performance, emphasizing the positive impact of nano-iron seed priming, particularly the green synthesized form at an optimal concentration of 50 ppm [42, 43]. Nano iron improves enzymatic activities involved in energy metabolism, leading to better seedling establishment and higher resilience to environmental stress as reported by as reported by [44–46]. Nanoparticles (NPs) have emerged as promising nanofertilizers because their nanoscale size, high surface-to-volume ratio, and tunable surface chemistry enable more efficient nutrient delivery compared to conventional fertilizers. Nanoscale carriers can improve the solubility and bioavailability of essential nutrients, reduce fixation or losses in soil, and ensure gradual and controlled release of nutrients aligned with plant demand [47, 48].

## Conclusion

This study indicates that biogenic iron nanoparticles synthesized using plant extracts as reducing and stabilizing agents offer a promising and eco-friendly approach for enhancing nutrient availability in pigeonpea. The nanoparticles displayed favorable nano-scale characteristics and stability, which likely contributed to improved solubility and bioavailability. Seed priming with these nanoparticles at 50 ppm showed measurable improvements in germination rate, shoot and root growth, and seedling biomass, along with a reduction in abnormal seedlings and non-viable seeds. These effects are consistent with enhanced nutrient uptake and early seedling metabolic activity. While the results are encouraging, further investigations are needed to validate these findings under field conditions, evaluate interactions with soil microbiota, and assess potential phytotoxicity, to ensure safe and effective application at larger scales.

## Data Availability

Data can be made available on request through email to corresponding authors.
